# AquaScouts: ePROs Implemented as a Serious Game for Children With Cancer to Support Palliative Care

**DOI:** 10.3389/fdgth.2021.730948

**Published:** 2021-12-08

**Authors:** Stefan Hoffmann, Robert Schraut, Thomas Kröll, Wiebke Scholz, Tatiana Belova, Johann Erhardt, Daniel Gollmer, Christian Mauck, Giorgos Zacharioudakis, Marcel Meyerheim, Panos Bonotis, Christine Kakalou, Maria Chatzimina, Christina Karamanidou, Annette Sander, Jana Didi, Norbert Graf, Pantelis Natsiavas

**Affiliations:** ^1^Serious Games Solutions, Promotion Software GmbH, Tübingen, Germany; ^2^Institute of Computer Science, Foundation for Research and Technology - Hellas, Heraklion, Greece; ^3^Clinic for Pediatric Hematology-Oncology, Saarland University Hospital and Saarland University Faculty of Medicine, Homburg, Germany; ^4^Institute of Applied Biosciences, Centre for Research and Technology Hellas, Thessaloniki, Greece; ^5^Department of Paediatric Haematology and Oncology, Hannover Medical School, Hannover, Germany; ^6^Centre for Molecular Medicine, Central European Institute of Technology, Masaryk University, Brno, Czechia

**Keywords:** childhood cancer, serious games, eHealth, mHealth, patient reported outcomes, palliative care

## Abstract

MyPal is a European initiative focusing on the use of the electronic patient reported outcome (ePRO) measures to enhance patient engagement in palliative cancer care via digital self-reporting palliative care for patients with cancer. As a part of its approach, MyPal also focuses on pediatric patients, implementing a specific digital health platform including a serious game to facilitate the reporting of the symptoms and overall status regarding their quality of life (QoL). To this end, the reduction of psychological burden related to frequent reporting, a.k.a. as “reporting fatigue” has been identified as a priority. In this study, we present the MyPal-CHILD platform, emphasizing on the serious game named AquaScouts and its key design decisions, while also emphasizing on the respective challenges. More specifically, we provide insights on the participatory design approach applied during the design of the platform and the high-level goals defined based on end-user input. In addition, the validation process applied before the use of the platform under real-world conditions is also presented. Finally, we discuss a number of challenges and the prospects of deploying eHealth interventions to support palliative care.

## Introduction

Although cancer in children is rare, it is the leading cause of death by disease past infancy. Diagnosis of cancer in a child is not only affecting the child *per se*, but also the peer's group (e.g., the family and the close circle of acquaintances as a whole). Beyond the disease itself, aggressive treatments with acute toxicities, prognostic uncertainties severely influence quality of life (QoL) of the patient. These strains are not only linked to the direct treatment phase, but may cause late effects and influence the well-being of survivors as well (1). The coping and comprehension of a cancer diagnosis substantially depend on the developmental stage of the child, which is associated with cognitive and emotional abilities and also the support provided from the social and family circle (2).

Palliative care for children is holistic supportive care and aims to start as soon as cancer is diagnosed, as there is evidence that the early integration of palliative care teams can significantly improve the outcome and QoL of patients and their caregivers (3). Such care needs to provide comprehensive support to both the child and the family considering the variety of physical, psychological, and spiritual aspects to cope with. To this end, palliative care should include both inpatient and outpatient settings and should not be restricted to end-of-life situations (4). It should be ideally provided by an experienced multidisciplinary team of health care professionals like oncologists, other specialized physicians, psychologists, nurses, social workers, etc.

The aim of the MyPal project is to exploit the recent advances in Information and Communication Technologies (ICT) by developing and offering a patient-centered digital health platform to support palliative care *via* the patient reported outcomes (PROs) paradigm by eliciting electronic means (ePRO approach). The MyPal platform has two branches, one for adults, i.e., MyPal-ADULT which is out of this article's scope, and one for children which is the focus of this paper. The MyPal-CHILD platform deploys “AquaScouts” as its main component, a serious game designed to collect input from patients. Pediatric patients between 6 and 17 years of age diagnosed with leukemia or a solid tumor are the main target end-user group of the MyPal-CHILD platform, emphasizing on reducing the psychological burden related to oncologic treatment and “reporting fatigue.”

Video games have already been used as an innovative method to collect data from children regarding health conditions, e.g., for obesity (5). Recently, FDA has approved the EndeavorRx video game as a therapeutic device to treat attention deficit hyperactivity disorder (6). Such approaches typically rely on an “entertaining” and attractive game to cause end-user engagement, and thus collect data from children and adolescents in an (almost) unobtrusive manner.

Interestingly, gamification has not yet been extensively applied for children palliative care (7, 8). Indeed, while several research and technical initiatives aim to support palliative care, very few of them engage in video games (9, 10) and only one of them was designed to support symptom reporting by patients in the context of palliative care (11). More specifically, Pain Squad+ presented by Jibb et al. (11) is a web-based smartphone application enabling spontaneous symptom reporting and the use of questionnaires to assess adolescent cancer pain. To encourage patient engagement, Pain Squad+ is based on a “gamified” approach where adolescents play the role of law-enforcement officers who receive rewards for adherence treatment and pain reporting.

The MyPal-CHILD gamification process includes a set of diverse features that enforce the motivational aspect of games (and implicitly the process of health status reporting), while also avoiding potential adverse effects (e.g., game addiction). In this study, we present the implementation of the MyPal-CHILD platform, emphasizing on the AquaScout serious game *per se*, also presenting the validation process applied before operating the platform in real-world conditions. Finally, several challenges and the prospects of deploying eHealth interventions to support palliative care are discussed.

## Methods

### Design

The AquaScout main design requirements were shaped as design “goals” by eliciting a usage scenario. The design goals presented here mainly reflect a high overview of user needs during the initial stages of the project design. Those goals were shaped based on literature, the project partners' previous experience and lessons learned from earlier attempts of the development of serious games, as well as their expertise in palliative care for children. To this end, healthcare professionals completed online surveys concerning the current palliative care context at their site, features of the intervention to be designed, how the system should behave, and their understanding regarding user needs. Additionally, six focus groups were conducted where “vignette” illustrations were used to elaborate on “usage scenarios” with patients. Each of the three clinical sites involved conducted one focus group with healthcare professionals involved in palliative care as well as one focus group with patients and their parents. Patients and parents had the chance to ask questions and brainstorm with clinicians and game designers providing their point of view on the system's behavior, attitude, and “must-have” functionalities. After the focus group, the patients completed a questionnaire that was evaluated by the consortium members. Both qualitative and quantitative data from the focus groups and the expert surveys were collected and aided in formulating the user “goals.”

Based on this process, the identified goals are summarized as follows:

G1. High long-time motivation to reduce dropout rates and increase the potential reporting of useful and health-relevant information.
G2. High retention, i.e., the ability to attract the end-user/player to voluntarily return and play the game multiple times.
G3. High sample rate of relevant symptoms reporting related to each patient's personal condition and clinical management needs.
G4. High quality and integrity of answers.
G5. As the “Game over” concept is also sometimes used metaphorically for death, the player should never fail and reach such a “Game over” state.
G6. As the end users/patients typically experience serious physical and psychological burden, the game should not require significant cognitive effort and it should encourage frequent engagement, but only for a short amount of time.

Based on these goals and the detailed elaboration on the “usage scenario” of the system a thorough list of technical requirements was produced, including functional, non-functional, and ethical requirements to guide the technical development of the platform.

Complimentary to the serious game app, a mobile app was developed to facilitate spontaneous symptom reporting and/or questionnaire answering by the informal patient care-giver (typically, one of the parents), sometimes also acting as a proxy in case the patient cannot for any reason answer the questionnaires.

Emphasizing on the need to collect valuable information in the context of the ePRO paradigm, well-defined standardized and validated questionnaires were implemented. More specifically, Mini-SSPedi (12) and SSPedi (13) questionnaires were digitally adapted to collect symptom information, PedSQL (14) to report QoL impact of the patient, European Quality of Life 5 Dimensions 3 Level Version (EQ-5D-3L) (15) to report the QoL impact of the parents, and the European Organization for Research and Treatment of Cancer satisfaction with cancer care core questionnaire (EORTC PATSAT C33) (16) to evaluate the satisfaction of the parents with the treatment of their child and the Impact of Family Scale (17). These questionnaires are asked as a part of the game or *via* the “MyPal Carer App” along the specific clinical study protocol and the main research endpoints, which are published by (18). It should be highlighted that MyPal Carer App is a simple questionnaire answering mobile app being used by the parent (acting as a proxy) only when the patient cannot report him/herself. This app also presents a collection of useful links for parents of cancer patients.

### Implementation

#### Technical Infrastructure

**Figure 1:**
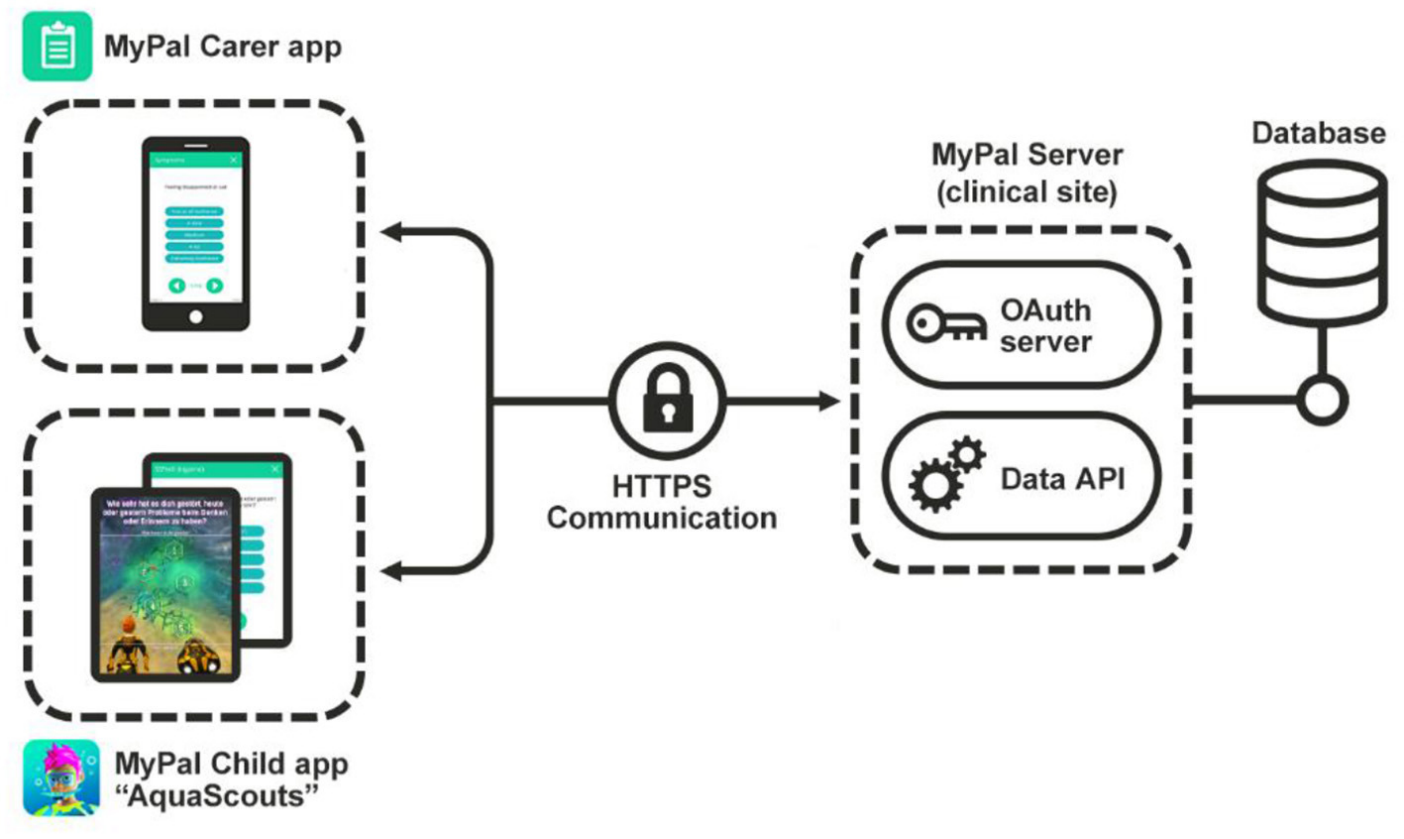
Technical overview: the MyPal Carer app is used by the caregiver to answer questionnaires as a proxy, while the pediatric patient can either answer them in-game *via* “AquaScouts” or by navigating to their own dedicated module in the MyPal Child app.

The two apps “MyPal Carer App,” “MyPal Child App”/“AquaScouts” were developed based on the “Unity” framework targeted for Android and iOS mobile devices. Besides the video game and the questionnaires, the mobile device's sensors are used to approximately estimate the number of daily steps. An overview of the MyPal-CHILD Technical Infrastructure is depicted in Figure 1. In terms of data flow, data collected in the mobile apps are sent to a REST API using SSL encryption and OAuth 2.0 authorization at a server in the clinical site that stores them to a local database.

#### The “AquaScouts” Serious Game

**Figure 2:**
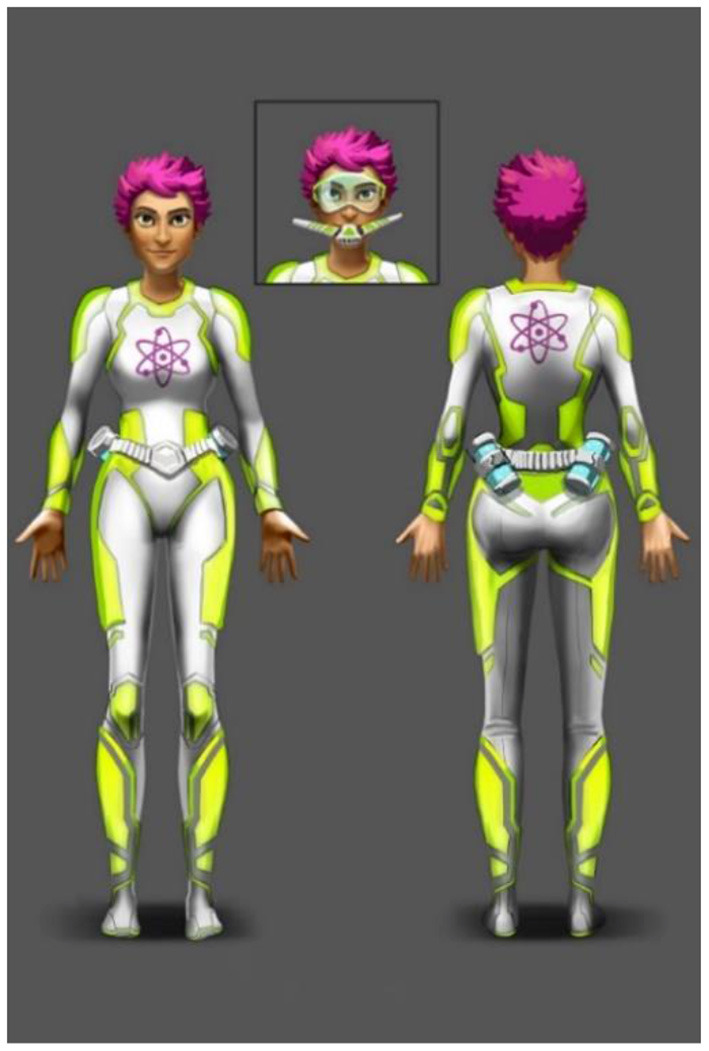
Diver concept drawing:- 2D concept drawings were used as a reference for the three-dimensional diver model. Players can choose a female or male diver character and customize their appearance.

**Figure 3:**
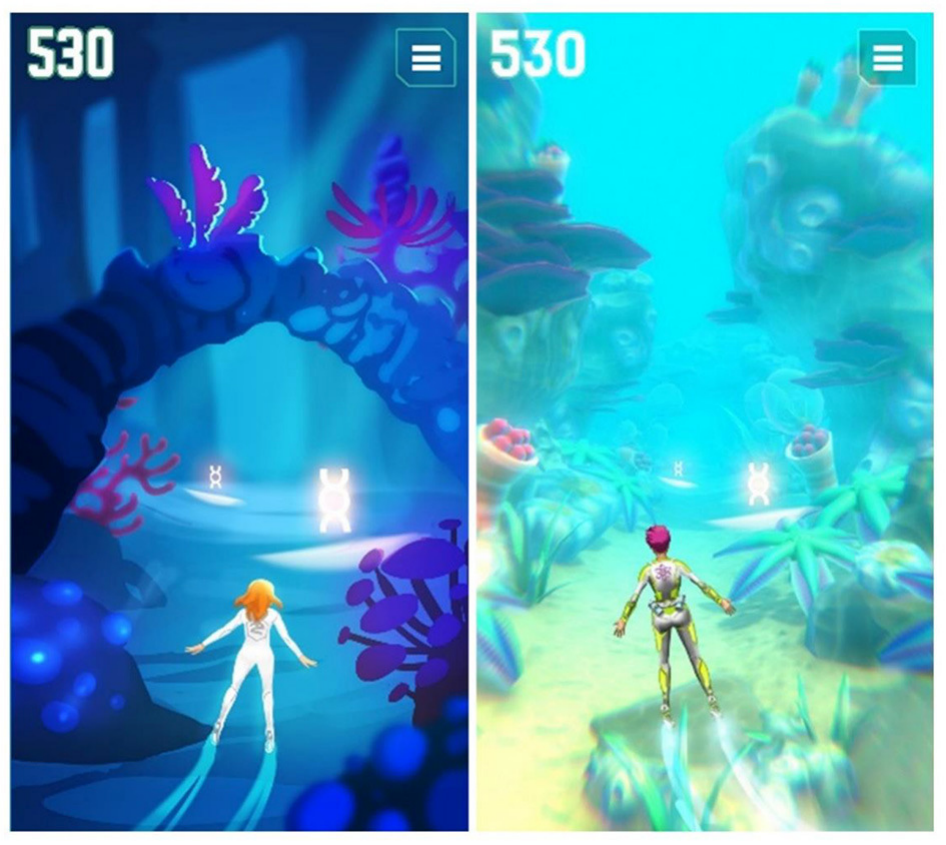
Visual style development of the underwater world.

To engage players right from the beginning, a compelling theme was created setting players as “AquaScouts,” i.e., scientific adventurers, who explore an alien ocean world. The player's avatar is a diver, who searches the seabed and tunnels of the world to collect artifacts, i.e., fragments of alien machinery, cultural heritage, and fossils of indigenous dinosaurs, as well as a non-indigenous parasite threatening the ecosystem. The esthetic focus is on the underwater aspect (wildlife, ancient civilization, corals rainbow fish, etc.), not emphasizing on the Sci-Fi aspect. The graphical style is as colorful as possible to create an enjoyable atmosphere. This setting was used to create a cheerful environment, which is appealing to all ages and genders. Since the target group of the study includes both children and adolescents, a suitable visual art style had to be defined that is accepted by the whole range of the children and adolescents age group. This applies not only to colors, shapes, and characters of the game world but also to the user interface design. As a result, a detailed and colorful 3D art style (Figure 2) was developed to bring the exotic underwater world to life (Figure 3).

**Figure 4:**
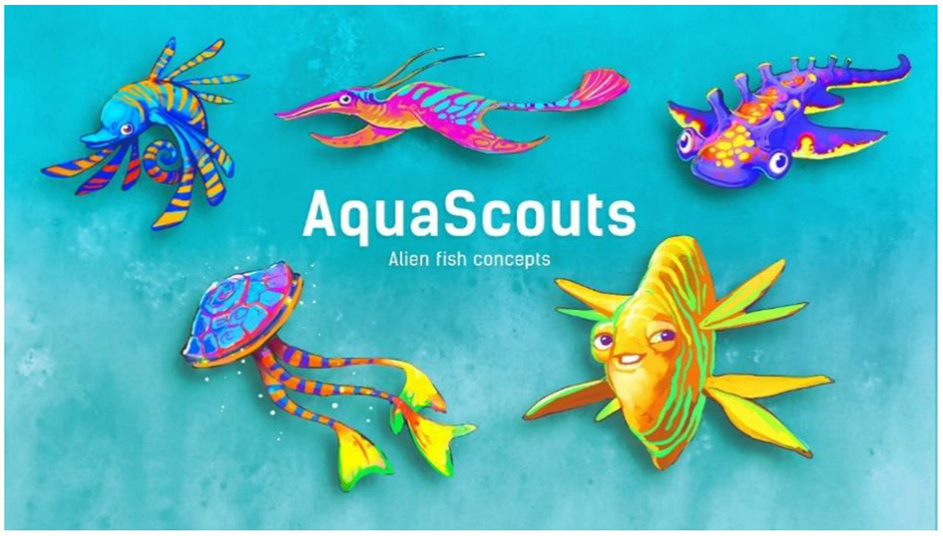
2D concepts of the alien creatures.

**Figure 5:**
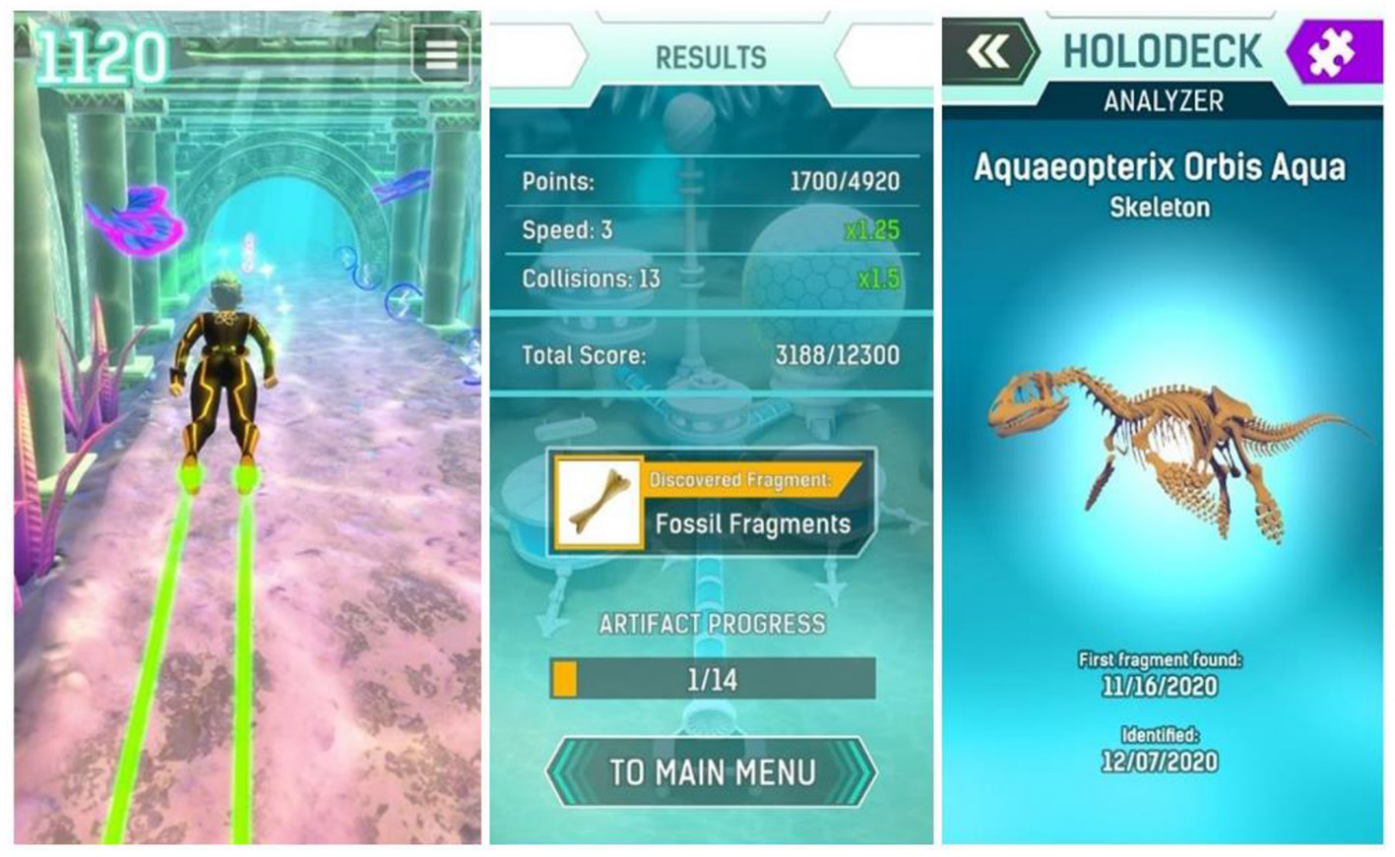
Left: Example of gameplay. Pink, blue and green blips can be collected for points. Obstacles need to be avoided. Center: Result screen with reward. Right: Exemplary finished artifact.

The ecosystem of the alien planet is bright and colorful, creating a beautiful and exciting atmosphere (Figure 4). The game itself is a “dexterity game” which gives the patient/player three challenges per day, each one aiming to last around 3 min (aiming to satisfy Goal 6—G6). As part of these challenges, players try to collect as many points as possible, curing parasitic infestation along the way. Additionally, there is a special reward when each challenge is completed for the first time. The special reward is always a fragment of a bigger artifact aiming to appeal to the human collecting instinct as part of a long-term motivation (G1 and G2). In order to satisfy Goal 5 (G5) it is not possible to fail a challenge or lose something (Figure 5). Challenges can be repeated to score a better result, but no additional special reward is given. Therefore, the highest incentivized course of action is to play three challenges of 3 min each per day aiming to avoid overindulgence (G6) as well as potential rejection by parents. To avoid questions being tapped away without any reflection (G4) a “long tap” (3 s) is required to answer a question. This time window is enough for a young patient to recognize the question being asked and, if the symptom is relevant, to abort a tap away attempt to switch to the honest answer.

**Figure 6:**
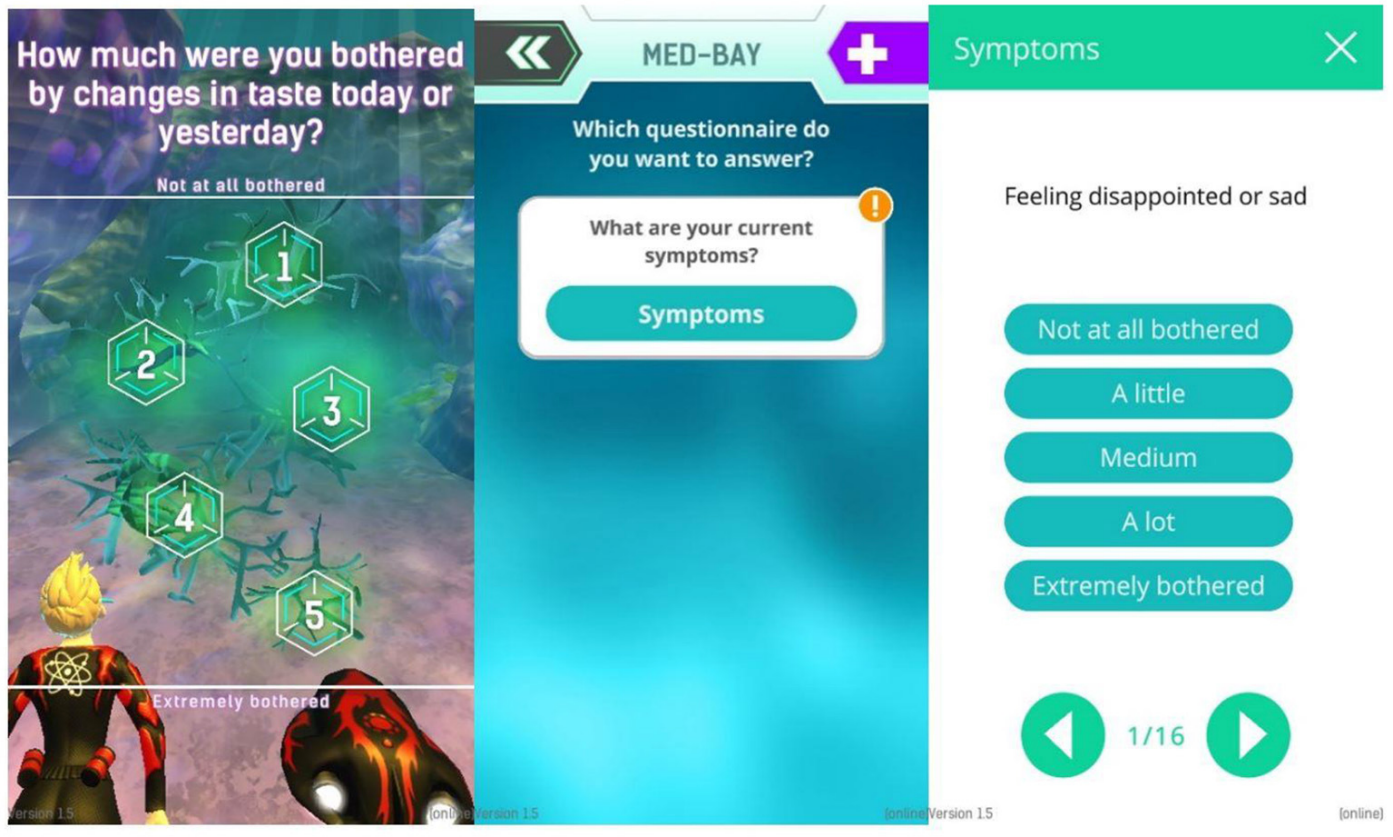
Left: In-game question, Center: Sick Bay with one questionnaire. More questionnaires can be shown here if available. Right: Exemplary symptom questionnaire.

For the in-game questions, the SSPEDI (created by Sung Lab) questionnaire was split up and its questions adapted for single use (Figure 6). This questionnaire was used as a model for the questions used in game because it is compact, easy to understand, but still checks on a good selection of symptoms. All questions adapted from SSPedi regarding symptoms are asked *via* the AquaScouts application, but the wording was changed to achieve better adaptation to “in-game” questioning. In total there are 15 different questions/symptoms dispersed throughout the challenges and only one question is asked at a time. All questions ask for the severity of specific symptoms (1: The symptom is not present. 5: The symptom is extremely strong). Indicatively, some question examples are: “How much were you bothered by headaches today or yesterday?,” “How much were you bothered by changes in how your body or face look today or yesterday?,” “How much were you bothered by pain (other than headache) today or yesterday?”

#### Questions Prioritization Scheme

In order to satisfy the goal of “personalization” (G3), the questions asked are prioritized based on the symptoms which have already been affecting the specific patient. Upon the answering of a question, depending on the severity of the reported symptom two things happen: First, the question is marked to a “cool-down” state which will ensure that the question is not asked again for a certain period of time. Second, the priority of the questions is adapted, depending on the reported severity of the symptom. The overall idea is that questions with high priority should be asked more frequently as they are more relevant. Besides the in-game questions, AquaScouts offers the option to fill out the questionnaire in the classic way of a digital questionnaire any time the patient desires to do so, also enabling uninterrupted gameplay.

Initially, all questions have a priority value of 50 and a complete questionnaire is answered to establish a baseline. The patient can answer each question by selecting the “severity” of a specific symptom, depicting how much he/she is affected by the respective symptom. Whenever a question is asked in the game, it will be the question with the highest priority, that is not in a cool down state. If all questions are in a cool down, then no questions are asked, and the patient can play with no interruptions.

The detailed cool-down and heat-up rules can be summarized as follows:

- If a question is answered with a severity of 1, the priority of the question is reduced by 30% (cool-down).
- If a question is answered with a severity of 2, the priority of the question is increased by +10 (heat-up).
- If a question is answered with a severity of 3, the priority of the question is increased by +15 (heat-up).
- If a question is answered with a severity of 4, the priority of the question is increased by +25 (heat-up).
- If a question is answered with a severity of 5, the priority of the question is increased by +40 (heat-up).
- Once per day all priorities are reduced by 20% to avoid the priority of severe symptoms to rise unchecked (heat-up balancing factor).

Beyond these rules acting upon the answers of the patients, to ensure all questions are asked regularly, the priority of all unanswered questions increases hourly. This increase gets stronger every day the question is not answered. The detailed time-related “heat-up” formula is: *PriorityIncreasePerHour* = *K* ^*^d with *K* serving as a balancing factor and *d* the number of days a question has not been answered.

#### MyPal Web Application for the Healthcare Professionals

The MyPal web application for healthcare professionals was created to serve as the entry point for physicians, nurses, social worker and psychologists. The web application is available in three languages German, Czech, and English (English are used only for development/demonstration purposes), and is responsive and accessible on smartphones, tablets, and desktop computers. It provides healthcare professionals with scalable and temporal visualization analytics, allowing them to comprehensively interpret the reported data and reinforce their actions by detecting significant differences in patients' symptoms and the quality of life in a timely fashion. The web application is designed to improve symptom management by enhancing patient-clinician communication using visual portrayal of information based on the ePRO questionnaire answers, which are collected in a real-time fashion, aiming to provide consistent health information along the disease timeline.

##### Aggregated Dashboard

**Figure 7:**
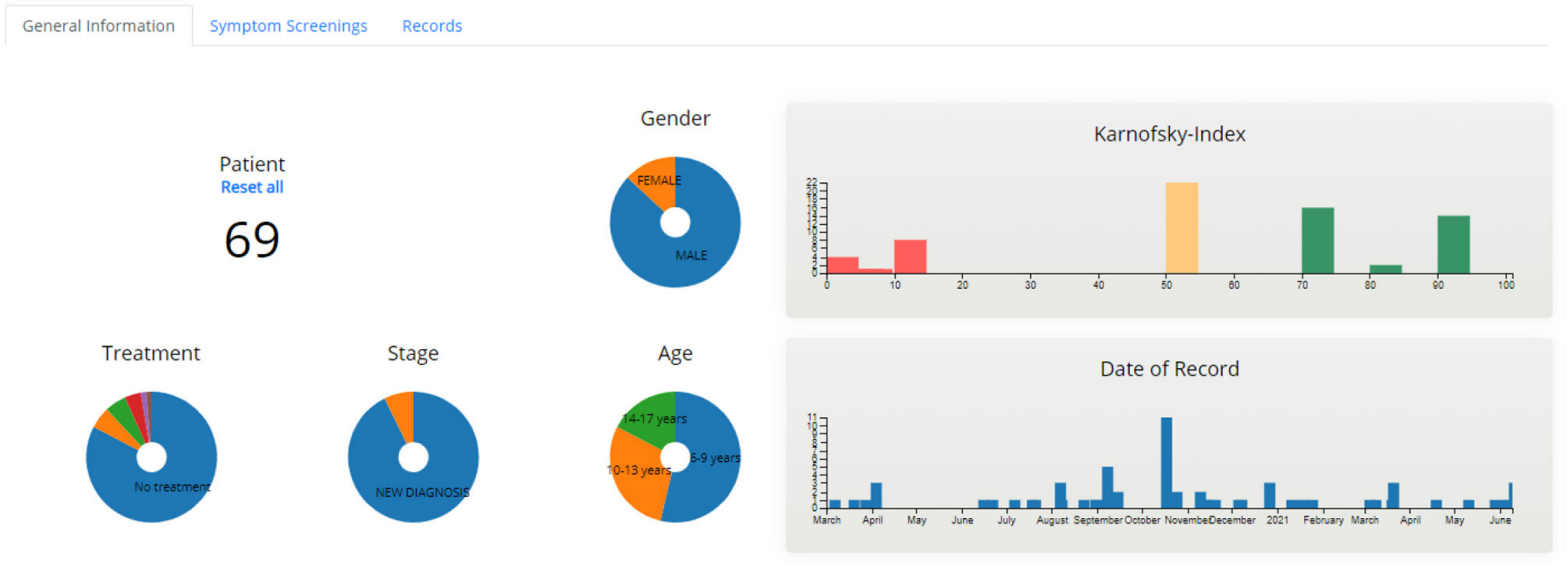
Aggregated Dashboard - General Information.

**Figure 8:**
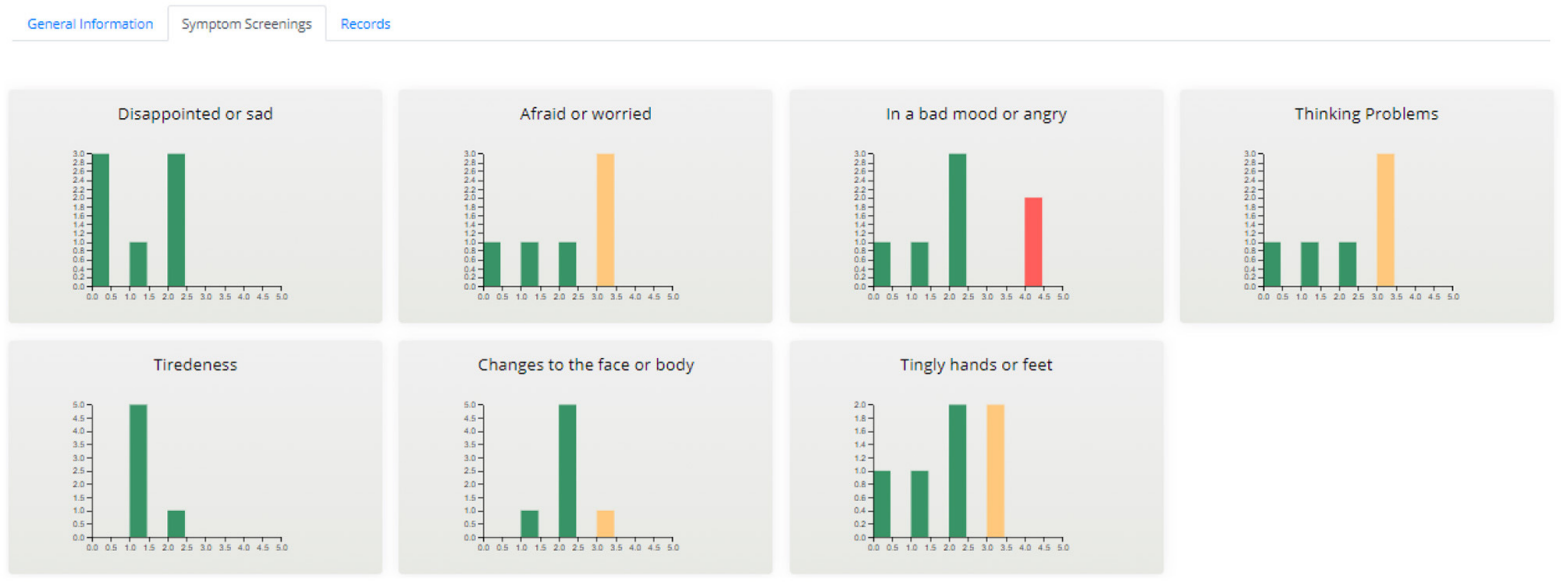
Aggregated Dashboard - Symptom Screenings.

The aggregated dashboard page (Figures 7, 8) uses interactive graphs to present information for all patients allocated to the specific healthcare professional. The visualized information includes demographics, treatment, stage of treatment, Karnofsky index, day of record, and scores of the completed PRO questionnaires. The healthcare professional is able to navigate and interact with the graphs and form patient groups by selecting age, gender, treatment, scores of PROs, etc., whereas abnormal scores are highlighted to provide a warning.

##### Individual's Dashboard

**Figure 9:**
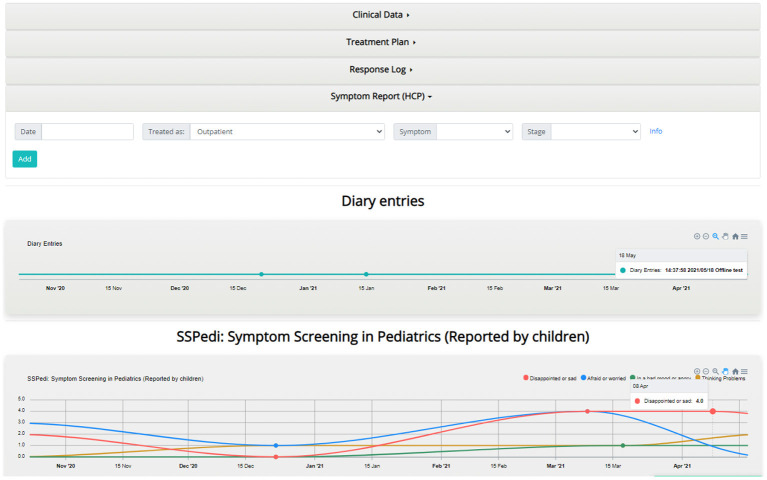
Individual's dashboard example.

The individual's dashboard presents information on each patient individually *via* interactive graphs and tables (Figure 9). PRO questionnaires scores and diary entries of patients are displayed as time-oriented data in graphs, which provide a variety of interaction techniques such as zooming, grouping, downloading, etc. The PRO scores displayed in graphs are classified based on whether they were reported by the patient or an informal patient caretaker (parents or clinicians). Furthermore, the healthcare professional is able to enter patient information such as treatment plans, symptoms, and clinician comments using forms, which are then displayed as part of the graphs or into tables.

Finally, the healthcare professional is able to easily access the administrator panel through the menu of the web application, as well as an imprint providing information on Personal Data Protection in accordance with the General Data Protection Regulation (EU) and a detailed tutorial of all the available techniques and views of the web application.

## Validation

### Validation Results From the Healthcare Professionals

Validation of the whole platform before being deployed in real-world conditions was identified as a key activity, engaging end-users (child patients, parents, and health care professionals) *via* focus groups, conducted both in physical presence and virtually (via teleconferences). More specifically, virtual validation workshops with healthcare professionals were hosted by one independent “validation expert” who was not involved in the development process of the system as one-on-one teleconference meetings focusing on the MyPal web application. Based on a scenario created for validation purposes (reflecting the “vignette” structure of the focus groups conducted during the design phase), specific tasks were presented to the healthcare professionals to perform while sharing their screens. A “think aloud” methodology was adopted to encourage the participants to express their initial reactions and thoughts unhindered, while the validation expert silently took notes. Each meeting lasted for ~1 h and was recorded for later analysis of end-user behavior, comments, and reactions. Every participant received in advance an email containing a presentation of the validation scenario and a consent form for the recording. Finally, after completing the validation tasks, the participants were asked to fill-in the widely accepted post-study system usability questionnaire (PSSUQ) to measure the perceived usability of the platform as well as some general questions regarding the demographics of the participant group, their self-reported computer literacy levels, and a couple of open-ended questions on the overall experience of using the web application. The results of this process were used to improve the acceptability and usability of the web application prior to its real-life use during the project pilot phase.

In advance of the initiation of the actual study, the developed mobile apps were tested within the framework of a 2-week rehearsal test phase at each of the three clinical sites involved. This rehearsal was carried out consistently according to previously jointly defined and shared guidelines: after setting up the required technical infrastructure, ~10 voluntarily consenting pediatric oncology ward staff members at each clinical site as well as their healthy children in the anticipated age range participated in the testing. They were provided with credentials for fake patient and caregiver accounts and, received introductory information on how to test and send back feedback in the form of completing a brief evaluation questionnaire and free-text comments after the end of the 2-week duration of testing. All feedback returned were evaluated and incorporated into further technical development and troubleshooting.

**Figure 10:**
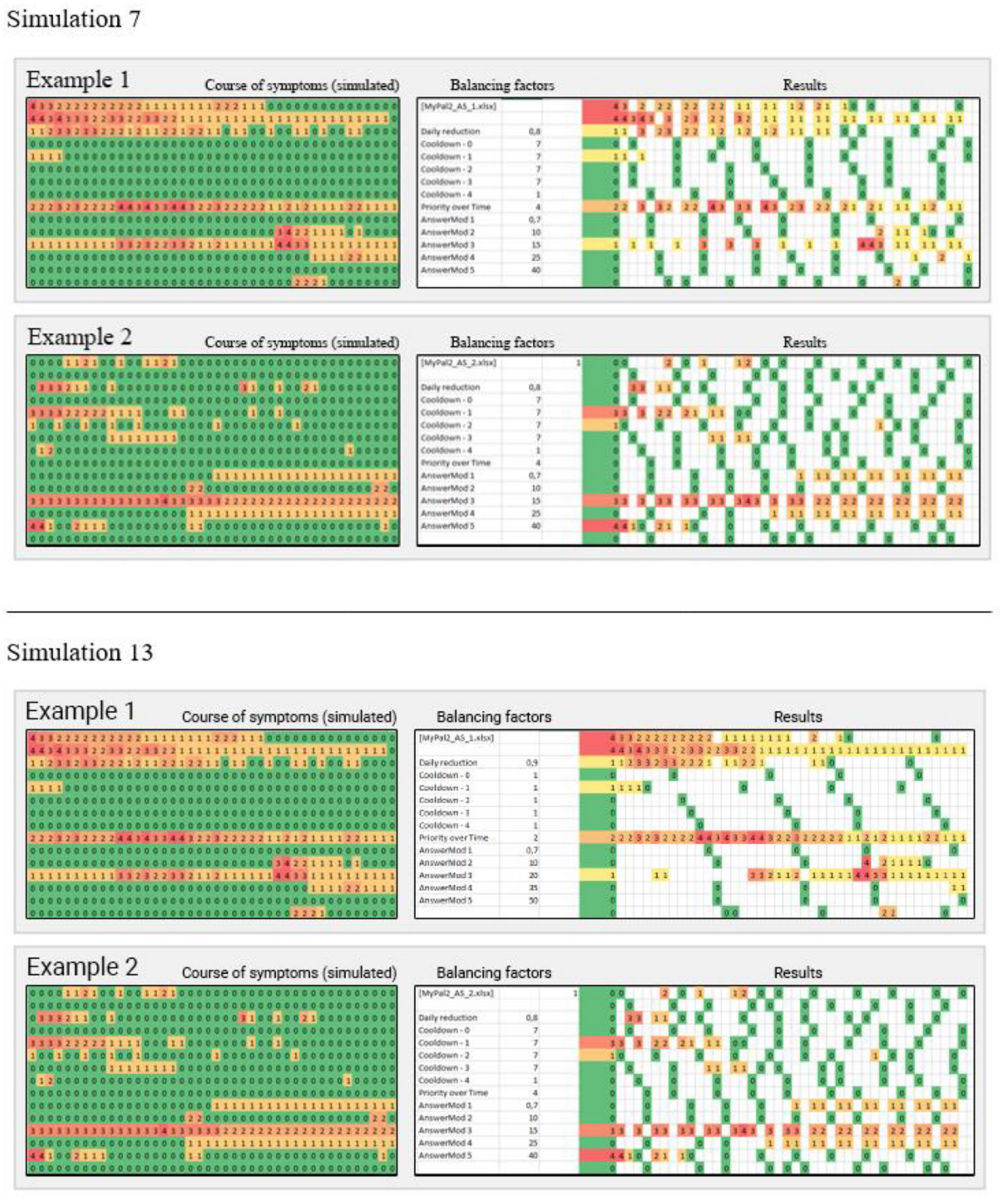
The patterns on the left show a simulated course of symptoms over 14 days, defined by clinicians. In the center are the balancing factors described in the “Questions Prioritization Scheme” section. To the right are the resulting sample patterns, assuming the app is used three times a day. Three columns represent 1 day (morning, noon, and evening).

In order to validate the question prioritization scheme, the clinical partners defined 18 exemplary scenarios for symptom reporting based on their experience and the specific patient cohort. This set of scenarios was used as input for 16 simulation runs of the presented priority algorithm, testing various combinations of heating-cooling balancing factors. An example of two scenarios from two different simulations is shown in Figure 10. In simulation 7, the symptoms with higher severities are sampled with a much higher frequency compared with simulation 13, but the sample rate of other symptoms is neglected. Respectively, in simulation 13 the sample rate of more relevant symptoms is still good enough, while the less severe symptoms are checked more often when compared with simulation 7.

### Current Status of the Studies

Currently, the presented platform is pilot tested in three European clinical sites as part of the MyPal-CHILD study (MyPal4Kids): the Saarland University Hospital as well as the Hannover Medical School in Germany and the University Hospital Brno in the Czech Republic. The study enrolment phase of children and adolescents with leukemia or solid cancers between 6 and 17 years and their parents was initiated in December 2020. While the COVID-19 pandemic inevitably led to challenges in terms of the clinical studies, as chronic patients were advised to avoid hospitals, still the MyPal-CHILD study proceeds (up to the 10th of October 2021, 58 pediatric oncology patients have been enrolled at the three clinical sites involved in the study)^1^.

## Discussion

Cancer in children and adolescents entails a series of challenges beyond physical symptoms, such as coping with fear and anxiety associated with a life-threatening disease and having to make difficult decisions about treatments. Timely communication of potential symptoms or condition worsening is of crucial importance, especially as this could lead to better clinical decisions. To this end, the ePRO paradigm might play an important role in terms of providing a more agile communication way which might reduce reporting fatigue and also a psychological burden, while also empowering patients to play a more central role in the overall decision-making progress.

Especially regarding children and adolescents, we argue that serious games could provide an important way to facilitate communication in the context of ePRO services. However, it should also be emphasized that the design and the development of such a game comes with a number of challenges, and as such, it is crucial that the context of the specific disease should be taken into account, and a patient-centered design approach should be applied.

In the context of MyPal initiative, an eHealth platform was developed using mobile apps and a serious game to support the application of the ePRO paradigm for pediatric palliative care, focusing on non-end-of-life situations. The proposed solution design focused on the end-user needs and identified a series of design goals to decrease potential reporting fatigue while also being able to collect useful information. The final design elaborated on a game-based approach using an adaptable algorithm to prioritize in-game questions. The overall system engages several well-defined questionnaires to evaluate several endpoints and it was validated in near real-world conditions before the actual clinical study. Currently, the MyPal4Kids clinical study is conducted in three clinical sites across Europe, aiming to evaluate the feasibility of the proposed approach.

In terms of limitations and challenges of the presented system based on the lessons learned so far, the risk of potential dropouts of end-users has been identified as the most crucial. Dropouts, i.e., the lack of long-term engagement with mobile apps in the context of mHealth has been clearly identified as an issue hindering the adoption of such technical solutions (19). While the pilot application in the clinical studies is still ongoing and therefore we cannot support it with data, we consider long-term engagement of patients the most important challenge regarding the adoption of mHealth solutions. Furthermore, while the MyPal consortium has selected to avoid claiming a CE mark for the respective software platform as if it was a medical device, we acknowledge the value of such a process and we identify it as a key potential future step.

As a whole, we argue that eHealth and the recent advances of ICT developments are already transforming the clinical research and clinical care settings, leading to new communication and procedural paradigms (Patient Reported Outcome Measures, Patient Reported Experience Measures, Decentralized Clinical Trials, etc.) based on emerging technologies which provide new capabilities in terms of functionality, information security, etc. As this paradigm shift is heavily dependent on constantly evolving technical developments, their practical applications and the development of specific tools raise cross-cutting issues regarding technical, healthcare, and psychological factors. For example, the usability and acceptability of mobile apps or games heavily depend on how their use might overlap with the respective clinical protocols or clinical research best practices. Similarly, information security issues might be directly related with patient safety risks. Thus, to face these issues, a new framework of systems and clinical processes design should be elaborated, engaging experts of various backgrounds in multidisciplinary teams. To this end, the systematic evaluation of eHealth applications via clinical studies is crucial, and as such, we argue that MyPal could provide useful insights for the integration of software apps in palliative care situations and beyond.

## Data Availability Statement

The original contributions presented in the study are included in the article/supplementary material, further inquiries can be directed to the corresponding author/s.

## Author Contributions

SH is the correspondent author and the coordinator of the AquaScouts and Carer app, with TK, RS, WS, DG, JE, TB, and CM contributing to the development process. CKar, MM, AS, and NG contributed in the design of the system, its validation and its pilot application. GZ developed the backend server for the applications needs, while MC developed the healthcare professionals' web application. CKak designed and applied the validation process. PB supported the design, implementation of the study, and acted as a technical liaison. CKar is the coordinator of the clinical studies and the project's clinical liaison. PN is the Technical Coordinator of the MyPal Project. All the authors actively contributed in writing the manuscript (PN led the manuscript authoring process) and reviewed and approved the content of the manuscript.

## Funding

The research was supported by the MyPal project which has received funding from the European Union's Horizon 2020 research and innovation program under the grant agreement No. 825872. This article reflects only the author's view. The Commission is not responsible for any use that may be made of the information it contains.

## Conflict of Interest

SH, RS, TK, WS, TB, JE, DG, and CM are employed by Serious Game Solutions, a division of Promotion Software GmbH, a software development company based in Tubingen, Germany. The remaining authors declare that the research was conducted in the absence of any commercial or financial relationships that could be construed as a potential conflict of interest.

## Publisher's Note

All claims expressed in this article are solely those of the authors and do not necessarily represent those of their affiliated organizations, or those of the publisher, the editors and the reviewers. Any product that may be evaluated in this article, or claim that may be made by its manufacturer, is not guaranteed or endorsed by the publisher.

## Footnotes

^1^By the end of March 2022, overall 100 study participants are anticipated to have been taken part in MyPal4Kids.
